# Establishing a National Molecular Surveillance Program for the Detection of *Plasmodium falciparum* Markers of Resistance to Antimalarial Drugs in Haiti

**DOI:** 10.4269/ajtmh.20-0833

**Published:** 2020-09-28

**Authors:** Karen E. S. Hamre, Baby Pierre, Ruth Namuyinga, Kimberly Mace, Eric W. Rogier, Venkatachalam Udhayakumar, Jacques Boncy, Jean Frantz Lemoine, Michelle A. Chang

**Affiliations:** 1 Division of Parasitic Diseases and Malaria, Center for Global Health, Centers for Disease Control and Prevention, Atlanta, Georgia;; 2 CDC Foundation, Atlanta, Georgia;; 3 Ministère de la Santé Publique et de la Population, Port-au-Prince, Haiti

## Abstract

Chloroquine remains the first-line treatment for uncomplicated malaria in Haiti, and until recently, sulfadoxine–pyrimethamine was the second-line treatment. A few studies have reported the presence of molecular markers for resistance in *Plasmodium falciparum* parasites, and in vivo therapeutic efficacy studies (TESs) have been limited. Recognizing the history of antimalarial resistance around the globe and the challenges of implementing TESs in low-endemic areas, the Ministry of Health established a surveillance program to detect molecular markers of antimalarial resistance in Haiti. Sentinel sites were purposefully selected in each of Haiti’s 10 administrative departments; an 11th site was selected in Grand’Anse, the department with the highest number of reported cases. Factors considered for site selection included the number of malaria cases identified, observed skills of laboratory technicians conducting rapid diagnostic tests (RDTs), stock and storage conditions of RDTs, accuracy of data reporting to the national surveillance system, and motivation to participate. Epidemiologic data from 2,437 patients who tested positive for malaria from March 2016 to December 2018 and consented to provide samples for molecular sequencing are presented here. Of these, 936 (38.4%) patients reported self-treatment with any medication since the onset of their illness before diagnosis; overall, 69 (2.8%) patients reported taking an antimalarial. Ten patients (0.4%) reported travel away from their home for at least one night in the month before diagnosis. Establishing a molecular surveillance program for antimalarial drug resistance proved practical and feasible in a resource-limited setting and will provide the evidence needed to make informed treatment policy decisions at the national level.

## INTRODUCTION

The island of Hispaniola remains the last malaria-endemic island in the Caribbean. Over 95% of reported cases on the island are from Haiti, where 21,998 confirmed cases were reported in 2016.^1^ Among these cases, over 99% were due to *Plasmodium falciparum*.^1^ Chloroquine (CQ) has been used to treat malaria in Haiti as either a monotherapy or combination drug since 1955.^2–4^ Despite widespread resistance to CQ worldwide, in the Caribbean and Central America, *P. falciparum* remains sensitive to CQ. A 3-day course of CQ plus a single dose of primaquine (PQ) (to target the gametocytes and prevent ongoing transmission) is the first-line treatment for uncomplicated malaria in Haiti.^1,5^ Until recently, the combination drug sulfadoxine–pyrimethamine (SP) was the second-line treatment for uncomplicated malaria in Haiti. (In April 2017, the artemisinin combination therapy [ACT] artemether–lumefantrine [AL] replaced SP as the second-line treatment.)^6^

The WHO recommends all national malaria programs to routinely monitor the therapeutic efficacy of the first- and second-line antimalarial drug combinations to inform the selection of effective treatments.^7^ With the emergence of CQ resistance, the WHO established a standardized therapeutic efficacy study (TES) protocol in 1964 to evaluate the in vivo response of *P. falciparum* to CQ.^8^ Since then, the protocol has undergone several revisions to incorporate both parasitological and clinical outcomes, additional drugs, and different transmission settings. The current established minimum length of follow-up for the standard TES is 28 or 42 days, depending on the half-life of the drug.^8^ Although TESs remain the gold standard for determining antimalarial drug efficacy, inherent limitations of conducting TESs in low-transmission settings exist, including the limited number of cases and potential attrition rates associated with the duration of follow-up.^8–10^ Molecular marker studies for the detection of genetic mutations of the parasites associated with resistance are an alternative method to monitor for antimalarial drug resistance, especially when a TES is not feasible. Specifically, point mutations at specific codons in genes involved in *P. falciparum* drug resistance serve as validated molecular markers of resistance to different antimalarial treatments.^8,11,12^ Molecular studies may be incorporated into routine surveillance to serve as an early warning system to inform recommendations for national malaria program strategies and treatment policies.^8^

In Haiti, in vivo TESs have been limited, although researchers began investigating the susceptibility of *P. falciparum* to CQ in the early 1980s because of concern about possible reduced susceptibility.^13^ Since then, the only TESs were conducted recently; the findings support the overall conclusions that *P. falciparum* remains highly sensitive to CQ in Haiti, but the monitoring of drug susceptibility should continue because of findings of a small number of reported treatment failures in each study.^9,10^ In addition, sporadic data have shown no presence of mutations in the *P. falciparum* Kelch 13 (K13) gene which may be responsible for resistance to artemisinin-based therapeutics.^14,15^ Over the years, molecular marker studies, conducted at irregular intervals and in geographic areas selected for convenience, present inconclusive results regarding the presence or absence of CQ- and/or SP-resistant haplotypes circulating in Haiti. Yet, they similarly conclude no change in treatment policy is currently warranted.^16–22^ These studies share limitations such as small sample sizes and limited geography.

Recognizing the history of antimalarial drug resistance around the globe, the challenges of implementing TESs in low-transmission settings, and the limitations of the previous studies conducted in Haiti, the Ministry of Health (MOH) established a standardized national molecular surveillance program to detect *P. falciparum* molecular markers of antimalarial drug resistance to inform policy recommendations. This article describes the creation of the program and reports on the epidemiologic factors associated with the malaria cases. Results from the initial laboratory analysis for sequencing of *P. falciparum* chloroquine resistance transporter (*Pfcrt*), *P. falciparum* dihydropteroate synthase (*Pfdhps*), and *P. falciparum* dihydrofolate reductase (*Pfdhfr*) to detect molecular markers of resistance to CQ, sulfadoxine, and pyrimethamine, respectively, are reported elsewhere.^23^

## MATERIALS AND METHODS

**Figure 1.**
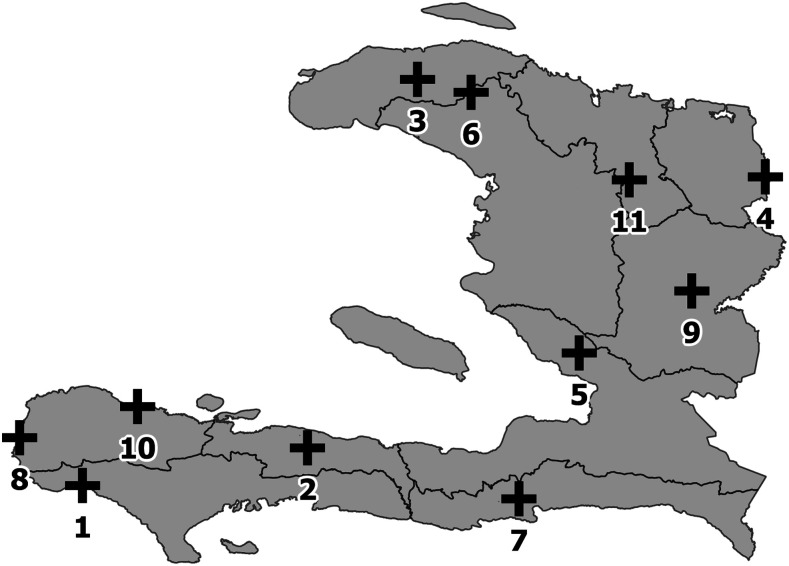
Location of 11 sentinel sites participating in the national molecular surveillance program in Haiti. Site name (Department): 1) Centre de Santé de Les Anglais (Sud), 2) Centre de Santé d’Arnaud (Nippes), 3) Centre de Santé de Beauchamp (Nord-Ouest), 4) Centre de Santé de Capotille (Nord-Est), 5) Centre de Santé de Cazale (Ouest), 6) Centre de Santé Clinique Jolivert (Artibonite), 7) Centre de Santé de Lavanneau (Sud-Est), 8) Centre de Santé de Saint-Hélène (Grand’Anse), 9) Centre de Santé de Thomonde (Centre), 10) Dispensaire Bon Samaritain des Roseaux (Grand’Anse), and 11) Dispensaire Saint Joseph de Pignon (Nord).

This molecular surveillance program was designed to integrate routine laboratory testing using existing staff and rapid diagnostic tests (RDTs) already supplied by the MOH. Additional resources for staffing, transportation for quarterly site visits, and per diem were added at the central level for one project manager to conduct surveillance system management, supervision, training, collection of samples, data analysis, and reporting. Staff from Haiti’s National Malaria Control Program (Programme National de Contrôle de la Malaria (PNCM)), with technical support from the U.S. CDC, conducted on-site visits to the three health facilities in each department that reported the highest RDT positivity rates, based on January 2014–June 2015 data from the national surveillance system. The purpose of each on-site visit was to evaluate the health facility to inform the selection of sentinel sites for Haiti’s national molecular surveillance program to monitor for resistance to antimalarial drugs. Several factors were considered for site selection, including 1) the number of malaria cases reported to the national surveillance system, 2) accuracy of data reporting to the national surveillance system as compared with laboratory registers, 3) observed skills of laboratory technicians performing RDTs, 4) stock and storage condition of RDTs, and 5) motivation to participate. Eleven health facilities, one in each of Haiti’s 10 administrative departments and one additional facility in Grand’Anse (the department with the highest number of reported cases at the time of selection), were purposefully selected to participate as sentinel sites (see Figure 1).

After site selection, the MOH led on-site trainings for the sentinel site staff responsible for consenting patients, testing patients with RDTs, and collecting surveillance data (i.e., laboratory technicians, nurses, nursing assistants, and clinicians, as specified by each site). Each training first covered the objectives of the program and the roles and responsibilities of the staff tasked with collecting dried blood spot (DBS) samples and surveillance form data. Next, a refresher training on performing finger pricks and RDTs, how to interpret RDT results, and Haiti’s malaria treatment policy was conducted. Timers were provided with the intended purpose to ensure the requisite time had passed before interpreting RDT results. This was followed by focused training on program-specific procedures to collect blood onto Whatman^®^ 903 filter papers (Sigma-Aldrich, St. Louis, MO), properly package the DBS samples for storage into individual Ziploc^®^ bags (SC Johnson, Racine, WI) with a single color-changing desiccant after 4–24 hours of drying time at room temperature, and complete the questionnaire and document responses on the surveillance forms. Emphasis was placed on collecting high-quality DBS samples, with examples of poor-quality DBS samples (e.g., insufficient blood to test; saturated, coagulated, or layered samples) illustrated. Sentinel site staff were trained on proper storage conditions, which included keeping the DBS samples at room temperature, away from sunlight, and adding a new desiccant if the color changed to pink before retrieval. Practical exercises were incorporated throughout the training for the sentinel site staff to practice all procedures. At the end of training, a workflow diagram of surveillance procedures was affixed on the wall of each site. The MOH provided each site with all materials required to conduct surveillance activities for an estimated 3–6 months, including unique barcoded patient identification numbers, surveillance forms, gloves, lancets, alcohol pads, cotton swabs, Whatman 903 filter papers, Ziploc bags, and desiccants. Materials were inventoried and replenished quarterly when surveillance forms and DBS samples were retrieved by the MOH project manager during scheduled supervisory visits.

Since March 2016, patients of all ages who present to a sentinel site with symptoms of malaria and who test positive by either RDT or microscopy during routine clinical care are eligible to participate. Existing sentinel site staff conduct the following activities without additional compensation: obtain informed consent for blood sample collection for molecular analysis, collect filter paper blood samples from consenting individuals, and complete the surveillance forms. Filter papers are stored at room temperature and retrieved quarterly for storage at the National Public Health Laboratory (Laboratoire National de Santé Publique (LNSP)). Filter papers are cut at the LNSP, with one section shipped to the CDC in Atlanta for molecular analysis. Surveillance forms are collected at the same time as the samples, and data are entered into an Epi Info™ (CDC, Atlanta, GA) database at the office of the PNCM. Demographics, residence information, history of present illness including any previous medications taken, and travel history data are collected. Descriptive and analytical statistics of the molecular surveillance form data were generated using Stata SE version 14 (Stata Corporation, College Station, TX); *P* values < 0.05 were considered statistically significant.

The protocol for molecular surveillance was approved by the Haitian Ministry of Public Health and Population Bioethics Committee as a non-research programmatic activity. This protocol was also reviewed by the CDC Center for Global Health and approved as a non-research surveillance activity. Blood specimens were collected only when participants (parents or guardians for children) consented to participate.

## RESULTS

**Figure 2.**
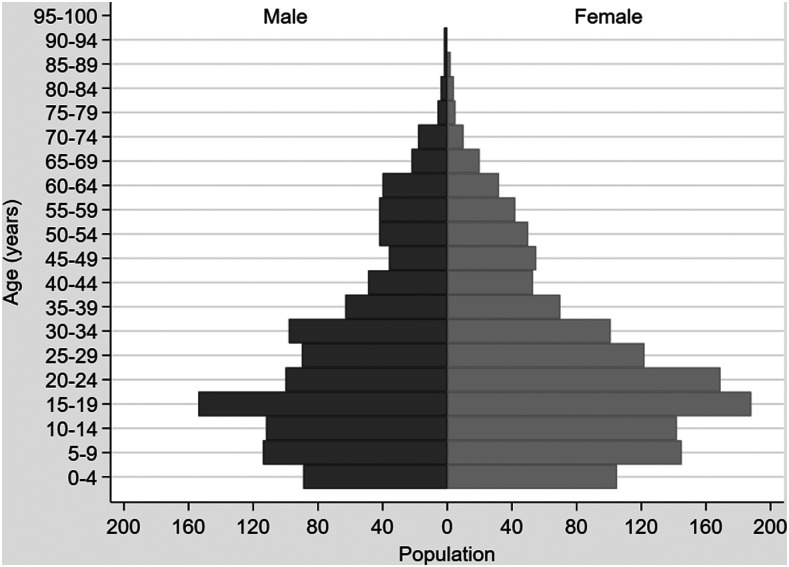
Population pyramid of 2,437 consenting patients who participated in Haiti’s national molecular surveillance program, by age category and gender, March 2016–December 2018.

Samples from 2,437 consenting individuals who tested positive for malaria by either RDT or microscopy were collected from the 11 sentinel sites from March 2016 to December 2018. This represents 40.1% of the 6,078 positive cases reported to the national surveillance system from these same 11 health facilities during this time period. The estimated percentages of cases with samples collected for participation in the molecular surveillance program ranged from 17.8% to 100% (Table 1). Of the 2,437 patients, all were Haitian residents and 2,222 (91.2%) sought care at health facilities within their home commune. Among the 215 individuals who sought care outside their home commune, 99 (46.0%) traveled to an adjacent commune as no health facility was located within their home commune. The median age of participants was 22.8 years (interquartile range [IQR]: 12.5–37), and 1,333 (54.7%) were female (Table 2). The age distribution of the patient population, by gender, is illustrated in Figure 2. The median duration of onset of symptoms before presenting to a health facility was 4 days (IQR: 3–5); this was consistent regardless of gender or age category (0 to < 5 years, 5 to < 15 years, 15 to < 65 years, and 65 years or older). Ten (0.4%) patients reported sleeping away from home for at least one night in the month before presenting to the health facility. The median duration of days away from home was 7.5 (IQR: 3–22). Six patients slept outside of their home commune, including three outside their home department. One of these patients lived in the Dominican Republic for 3 years before returning to Haiti, and 2 days later tested positive for malaria at the health facility. At the time of presentation, this patient had been experiencing symptoms for 10 days.

**Table 1:** Numbers of persons consented for molecular surveillance program with samples collected and cases reported to national surveillance, with estimated percentages, by health facility, March 2016–December 2018

Health facility	Department	Number of persons consented with samples collected	Number of cases reported to national surveillance	Estimated* % of reported cases with samples collected
1) Centre de Santé de Les Anglais	Sud	1,066	2,890	36.9
2) Centre de Santé d’Arnaud	Nippes	358	493	72.6
3) Centre de Santé de Beauchamp	Nord-Ouest	1	0	100†
4) Centre de Santé de Capotille	Nord-Est	13	31	41.9
5) Centre de Santé de Cazale	Ouest	83	95	87.4
6) Centre de Santé Clinique Jolivert	Artibonite	1	0	100†
7) Centre de Santé de Lavanneau	Sud-Est	12	16	75.0
8) Centre de Santé de Saint-Hélène	Grand’Anse	536	595	90.4
9) Centre de Santé de Thomonde	Centre	22	26	84.6
10) Dispensaire Bon Samaritain des Roseaux	Grand’Anse	343	1,930	17.8
11) Dispensaire Saint Joseph de Pignon	Nord	2	2	100
Total	–	2,437	6,078	40.1

* Percentages are estimated as the column data come from different sources (i.e., the number of persons consented with samples collected is not a true subset of the number of cases reported to national surveillance).

† Although 0 cases were reported to national surveillance, it is estimated the one positive case with a sample collected for the molecular surveillance program was the only case during this time period.

**Table 2:** Patient characteristics and surveillance form data from 2,437 patients who consented to participate in Haiti’s national molecular surveillance program, March 2016–December 2018

Surveillance form data	*N* = 2,437, *n* (%) or median (interquartile range)
Gender, female	1,333 (54.7)
Age (years)	22.8 (12.5–37)
Age category (years)	
0 to < 5	194 (8.0)
5 to < 15	513 (21.1)
15 to < 65	1,598 (65.6)
65 and older	96 (3.9)
Missing	36 (1.5)
Duration of symptoms before diagnosis (days)	4 (3–5)
Took any medication since the onset of illness before diagnosis	936 (38.4)
Took antimalarial medication since the onset of illness before diagnosis	69 (2.8)
Slept away from home ≥ 1 night in the previous month	10 (0.4)
Duration of travel away from home (days)	7.5 (3–22)

Over one-third (38.4%; *N* = 936) of patients reported taking any medication (i.e., self-treated) after the onset of symptoms and before diagnosis at the health facility. Individuals who had self-treated were more likely female than male (*P* < 0.001), were older (*P* < 0.05), and had longer duration (days) of symptoms before diagnosis (*P* < 0.001) than those who did not take any medications before seeking care (Table 3). Most individuals who had self-treated took acetaminophen (*n* = 490) or a nonsteroidal anti-inflammatory drug (*n* = 453) before diagnosis, 69 took an antimalarial drug, and 15 took an antibiotic (Table 4). All but one patient who reported taking an antimalarial took CQ. No differences in gender, age, duration of symptoms before seeking care, or whether care was sought outside the home commune were found between patients who reported self-treatment by antimalarial drug before diagnosis and those who did not (data not shown). Although over 50% of patients self-treated with any medication before diagnosis at four of the 11 sentinel sites, self-treatment with antimalarial medications was generally uncommon (Table 5). In two health facilities, more than 5% of people reported self-treatment with an antimalarial (35/536 [6.5%] in Centre de Santé de Saint-Hélène and 2/13 [15.4%] in Center de Santé de Capotille).

**Table 3:** Characteristics of patients, by reported self-treatment status, before malaria diagnosis at the sentinel site, March 2016–December 2018

Characteristic	Self-treatment (any medication), *n* (%) or median (interquartile range)		*P*-value*
	Yes (*N* = 936)	No (*N* = 1,501)	
Gender†			< 0.001
Female	555 (59.3)	778 (51.8)	
Male	379 (40.5)	718 (47.8)	
Age (years)	23.9 (14.5–38.0)	21.6 (11.6–37.0)	< 0.05
Age category (years)‡			Overall < 0.01
0 to < 5	61 (6.5)	133 (8.9)	Reference
5 to < 15	180 (19.2)	333 (22.2)	0.36
15 to < 65	657 (70.2)	941 (62.7)	0.01
65 and older	30 (3.2)	66 (4.4)	0.97
Duration of symptoms before diagnosis (days)	4 (3–6)	4 (3–5)	< 0.001
Sought care outside home commune			0.09
Yes	71 (7.6)	144 (9.6)	
No	865 (92.4)	1,357 (90.4)	

* Analyzed using Pearson’s chi-square test for categorical variables or the Wilcoxon rank-sum test for continuous variables.

† There were seven patients with missing gender data excluded from analysis.

‡ There were 36 patients with missing age data excluded from analysis.

**Table 4:** Category of medications taken among patients who reported self-treatment before diagnosis

Medication category	*N* = 936, *n* (%)
Antimalarial*	69 (7.4)
Acetaminophen	490 (52.4)
Nonsteroidal anti-inflammatory drug	453 (48.4)
Antibiotic†	15 (1.6)
Other	11 (1.2)

CQ = chloroquine.

* Antimalarials reported: CQ (*n* = 68), sulfadoxine–pyrimethamine (*n* = 1), and mefloquine (*n* = 1). (Note: one subject reported taking both CQ and mefloquine before diagnosis.)

† Antibiotics reported: amoxicillin (*n* = 12), ampicillin (*n* = 2), and azithromycin (*n* = 1).

**Table 5:** Numbers and percentages of patients who reported self-treatment by any medication, and by an antimalarial, before malaria diagnosis, by sentinel site, March 2016–December 2018

Sentinel site	*N*	Self-treatment (any medication)	Self-treatment (antimalarial)
		Yes, *n* (%)	Yes, *n* (%)
1) Centre de Santé de Les Anglais	1,066	264 (24.8)	18 (1.7)
2) Centre de Santé d’Arnaud	358	254 (71.0)	0
3) Centre de Santé de Beauchamp	1	0	0
4) Centre de Santé de Capotille	13	8 (61.5)	2 (15.4)
5) Centre de Santé de Cazale	83	47 (56.6)	1 (1.2)
6) Centre de Santé Clinique Jolivert	1	0	0
7) Centre de Santé de Lavanneau	12	1 (8.3)	0
8) Centre de Santé de Saint-Hélène	536	227 (42.4)	35 (6.5)
9) Centre de Santé de Thomonde	22	16 (72.7)	0
10) Dispensaire Bon Samaritain des Roseaux	343	119 (34.7)	13 (3.8)
11) Dispensaire Saint Joseph de Pignon	2	0	0

Associated costs of the molecular surveillance program include materials for collecting blood spots (filter paper, lancet, storage bags/desiccant, gloves; $2 per sample) and molecular sequencing for CQ-resistant *Pfcrt* and SP-resistant *Pfdhfr* and *Pfdhps* mutations ($40 per sample for three genes). Both the real-time PCR machine for species-specific analysis at the LNSP and the genetic sequencer at the CDC were already available.

For the initial publication of molecular drug resistance markers from samples collected from 2016 to 2017, no evidence for CQ resistance markers was found (for more details, see Rogier et al.^23^). Parasite strains possessing markers of resistance (mutations in multiple codons involving *Pfdhfr* and *Pfdhps* genes) to SP were very rare, with the exception of the S108N mutation in *Pfdhfr*, which was detected in 47% of patients. Only one isolate (0.1% of total sequenced) was found to have multiple codon mutations in looking at both *Pfdhfr* and *Pfdhps*. As AL use was not recommended in Haiti until April 2017, K13 sequencing was not performed for the initial report. However, K13 sequencing is now integrated into molecular marker investigations from specimens.

## DISCUSSION

Establishing Haiti’s national molecular surveillance program proved practical and feasible with additional investments. Existing health facility staff at the sentinel sites voluntarily completed the activities required to collect and report data and samples to the national molecular surveillance program. One aspect of the program which is still deficient is the necessity for samples to be sent outside Haiti for gene sequencing. Capacity for higher level genomic assays does not currently exist at the LNSP.

As expected, the majority of samples were collected from the four sentinel sites located in the three departments (Grand’Anse, Nippes, and Sud) in the southwestern peninsula of Haiti where the highest incidences of malaria are reported (22.3/1,000; 4.4/1,000; and 2.5/1,000 in 2016, respectively) (MOH, unpublished data). A limitation is that not all cases reported to national surveillance from the sentinel sites consented to participate in the molecular surveillance program and have their samples tested. One potential obstacle to participation is that the molecular surveillance program requires a second finger prick to collect the DBS sample after the initial RDT is performed and the patient tests positive for malaria. The second finger prick could potentially be eliminated if parasite DNA was extracted from the RDT strip, as has been successful in other settings.^24–26^ Still, some sites have higher estimated participation than others. Comparing the two Grand’Anse sentinel sites, Centre de Santé de Saint-Hélène consented an estimated 90.1% of cases, whereas Dispensaire Bon Samaritain des Roseaux consented an estimated 17.8% of cases. The latter site had reportedly high staff turnover, with new staff requiring training during regularly scheduled quarterly supervision and collection visits, as well as a higher number of positive cases. Sites with a higher number of cases will have a higher overall burden in terms of total time to consent patients, administer the questionnaires, draw the blood samples, and package the DBS samples for storage. Consenting to a second finger prick also requires the trust of the provider, and high turnover may limit that trust. Motivation to participate, one of the initial site selection criteria, may change with changing personnel.

Self-treatment for symptoms of malaria is an established problem worldwide, in part due to historically pervasive presumptive treatment practices.^27–31^ Haiti is no exception; in one study at a single health facility in October 1995, 39.2% of patients presenting with symptoms of malaria were found to have detectable levels of CQ in their blood.^32^ This is higher than the 2.8% of patients who reported self-treatment with an antimalarial to the molecular surveillance program, although blood levels for CQ were not assessed. The reduction in self-treatment with antimalarials may in part be due to Haiti’s 2012 revised diagnosis and treatment guidelines, which added recommendations to confirm all suspected cases of malaria by parasitological diagnostic test before treatment.^33^ Yet, antimalarials and other medications are still widely available in Haiti and can be purchased over the counter at pharmacies and shops, with no requirement for testing.^34^ Regional variation with respect to access to commercial medication vendors across Haiti may also have influenced self-treatment reported across sites. Still, in the two molecular surveillance program health facilities located in Grand’Anse, 6.5% and 3.8% of patients reported self-treatment with an antimalarial before diagnosis, indicating antimalarials are available and being used for self-treatment in the department with the highest reported incidence nationwide. Although an inherent incentive exists for patients with symptoms of malaria to seek care as diagnostic tests for malaria are free in health facilities, and those who test positive receive free treatment as per the MOH guidelines, access to care remains limited. In an effort to improve access, in February 2018, Haiti modified its treatment policy to allow community health workers to test and treat for malaria.^35^ As of December 2018, more than 2,200 new community health workers have been trained and placed nationwide (MOH, unpublished data). Future analyses should evaluate whether these changes have had an effect on reported self-treatment rates, or if targeted educational campaigns are needed.

While TESs remain the gold standard for determining antimalarial drug efficacy, and the overall annual number of cases in Haiti are sufficient to conduct TESs (21,998 confirmed cases were reported in 2016), a TES has been challenging to conduct in Haiti.^1^ A difficulty in conducting TESs is the long duration of monitoring required of 28 or 42 days, depending on the half-life of the drug, which can lead to losses to follow-up.^8^ This was evident in two recent TESs conducted in Haiti with observed high losses to follow-up; one study reported 19/107 (17.8%) at day 7, 30/107 (28.0%) at day 14, 39/107 (36.4%) at day 28, and 47/107 (43.9%) at day 42.^9^ The other study reported inconsistent monitoring with 30/61 (49.2%) monitored on day 3, 28/61 (45.9%) on day 7, 13/61 (21.3%) on day 14, 18/61 (29.5%) on day 21, and 33/61 (54.1%) on day 28 at the end of the study.^10^

Molecular marker studies provide a useful alternative to TESs in logistically challenging low-transmission settings and are useful for surveillance. Dried blood spot samples can be easily prepared, stored, and transported. Molecular markers for several different antimalarial medications can be characterized using a single filter paper. Haiti’s molecular surveillance program specifically tests for molecular markers to CQ, sulfadoxine, and pyrimethamine but will begin to test for mutations in the K13 gene with the transition to AL use in April 2017. Monitoring for antimalarial drug resistance at the national level is especially important in nations still using first- and second-line treatments to which widespread resistance has developed in other parts of the world, and using molecular surveillance is feasible for drug resistance markers that are well-characterized, such as for CQ, SP, and ACTs. Honduras, like Haiti, is on the path toward malaria elimination and treats uncomplicated malaria with CQ. In 2010, the Honduran MOH developed a national surveillance program to sequence *Pfcrt* to detect molecular markers of resistance to CQ.^36^ If different antimalarial drug combinations are used in Haiti in the future, the molecular surveillance program can monitor for other putative genetic markers of resistance. Lack of CQ resistance markers and highly resistant SP genotypes in Haiti reported by the data from the 2016–2017 samples suggests that treatment policies at the time were reasonable.^23^ Integration of K13 markers in future sequencing efforts will ensure the updated treatment policy will remain appropriate for Haiti.

Recently, there is renewed commitment to accelerate the island of Hispaniola toward malaria elimination to create a malaria-free zone across the Caribbean.^5^ As part of the approach, in October–November 2018, the MOH in partnership with Malaria Zero (https://www.malariazeroalliance.org/) piloted a targeted mass drug administration (MDA) campaign in Grand’Anse department that reached more than 36,000 individuals.^37^ Data collected from the molecular surveillance program helped inform the selection of SP for this targeted MDA campaign where directly observed treatment of SP and a single low-dose PQ were administered to all eligible residents. Haiti’s National Strategic Plan for 2016–2022 includes the potential for additional and/or expanded rounds of MDA in areas approaching interruption in line with the WHO’s recommendations.^38,39^ Although existing data suggest that no change is needed to the national treatment policy, surveillance to monitor and detect the emergence of markers of antimalarial resistance will be critical in areas under selected drug pressure where MDA is implemented.^23^ The continuation of the molecular surveillance program will provide the evidence needed to make informed treatment policy decisions at the national level.
